# Phytochemical and Preliminary Biological Evaluation of *Acanthus balcanicus* Aqueous Extract in Streptozotocin-Induced Diabetes

**DOI:** 10.3390/ph19071088

**Published:** 2026-07-15

**Authors:** Denisa Floriana Vasilica Pirscoveanu, Cristian Cosmin Arsenie, Diana-Maria Trasca, Adina Kamal, Cristina Popescu, Carmen Vladulescu, Ion Dorin Pluta, Renata Maria Varut, Rodica Dirnu, Maria Stoica, Daniela Cîrțînă, Gabriela Pura

**Affiliations:** 1 Department of Neurology, Faculty of Medicine, University of Medicine and Pharmacy of Craiova, 200349 Craiova, Romania; 2 Discipline of Anatomy, Department of Anatomy, University of Medicine and Pharmacy, 200349 Craiova, Romania; 3 Department of Internal Medicine, University of Medicine and Pharmacy of Craiova, 200349 Craiova, Romania; diana.trasca@umfcv.ro (D.-M.T.);; 4 Faculty of Horticulture, Department of Biology and Environmental Engineering, University of Craiova, 200585 Craiova, Romania; 5 Faculty of Medical and Behavioral Sciences, Constantin Brâncuși University of Târgu Jiu, 210185 Targu Jiu, Romania; 6 Research Methodology Department, Faculty of Pharmacy, University of Medicine and Pharmacy of Craiova, 200349 Craiova, Romania; 7 Department of Intensive Care and Anesthesia, Emergency County Hospital, 200349 Craiova, Romania; 8 Department of Medical Devices and Pharmaceutical Practice, Iuliu Hațieganu University of Medicine and Pharmacy, 400012 Cluj-Napoca, Romania

**Keywords:** diabetes mellitus, *Acanthus balcanicus*, *Vaccinium myrtillus*, phenolic compounds, streptozotocin, oxidative stress

## Abstract

**Background/Objectives**: Diabetes mellitus is a multifactorial metabolic disorder characterized by chronic hyperglycemia, oxidative stress, and lipid metabolism dysregulation, leading to severe systemic complications. Increasing interest has been directed toward plant-derived bioactive compounds as potential therapeutic alternatives. The present study aimed to comparatively evaluate the phytochemical profile and biological effects of *Acanthus balcanicus* and *Vaccinium myrtillus* in a streptozotocin-induced experimental model of diabetes mellitus. **Methods**: Aqueous extracts of both plant species were prepared and characterized using high-performance liquid chromatography and spectrophotometric assays to determine phenolic and flavonoid content. Antioxidant activity was assessed using the DPPH radical scavenging method. In vivo experiments were conducted on streptozotocin-induced diabetic mice over a five-week period, evaluating glycemic levels, lipid profile, body weight, food and water intake, and oxidative stress markers, including SOD, GPx, GR, and lipid peroxidation. **Results**: Both extracts exhibited antioxidant activity and contained measurable amounts of phenolic compounds and flavonoids. *Acanthus balcanicus* demonstrated higher radical scavenging capacity, whereas *Vaccinium myrtillus* showed higher total flavonoid content. In vivo, administration of the plant extracts was associated with improvements in selected metabolic parameters compared with untreated diabetic animals. In particular, *Acanthus balcanicus* produced a marked reduction in blood glucose levels and improved lipid profile and oxidative stress markers under the present experimental conditions. Both extracts reduced hyperglycemia, hyperlipidemia, and oxidative stress markers compared with the diabetic control group. **Conclusions**: The findings provide preliminary evidence that aqueous extracts of *Acanthus balcanicus* and *Vaccinium myrtillus* may exert glucose-lowering and antioxidant effects in a streptozotocin-induced experimental model of diabetes. Under the present experimental conditions, administration of *Acanthus balcanicus* was associated with greater improvement in selected metabolic and oxidative stress parameters than the botanical comparator *Vaccinium myrtillus*. However, these findings remain preliminary and require confirmation in dose–response studies including a standard pharmacological control. However, these results should be interpreted cautiously because the study was conducted in a short-term animal model and did not include a positive pharmacological control or direct mechanistic validation. Further studies including standard antidiabetic drugs, insulin measurements, extended biochemical and histological evaluation, and mechanistic assays are required before any therapeutic relevance for diabetes management can be established.

## 1. Introduction

Diabetes mellitus is one of the most prevalent and severe chronic multisystemic disorders, with a continuously increasing global incidence and prevalence, and it remains a major cause of early morbidity and mortality. It is a complex metabolic disease of multifactorial etiology, characterized by persistent hyperglycemia resulting from impaired insulin secretion, insulin resistance, or a combination of both. These alterations lead to profound disturbances in carbohydrate, lipid, protein, and hydroelectrolytic metabolism, ultimately contributing to the development of long-term complications affecting the eyes, kidneys, peripheral nervous system, and vascular structures [1,2,3].

At the cellular level, diabetes mellitus is associated with significant metabolic dysregulation, as glucose is inadequately utilized for energy production, leading to depletion of hepatic glycogen reserves and enhanced mobilization of alternative energy substrates. Consequently, lipid metabolism becomes altered, resulting in elevated circulating triglycerides and an imbalance in lipoprotein fractions, typically characterized by decreased high-density lipoprotein (HDL) cholesterol and increased low-density lipoprotein (LDL) cholesterol levels. These changes contribute to the development of atherosclerosis and increase the risk of cardiovascular complications. In addition, chronic hyperglycemia induces oxidative stress through the overproduction of reactive oxygen species, which further exacerbates cellular damage and plays a central role in the progression of diabetic complications [4,5,6].

Although conventional antidiabetic therapies, including insulin and oral hypoglycemic agents, are effective in controlling blood glucose levels, their long-term use is often associated with adverse effects, high costs, and limited accessibility in certain populations. As a result, there has been increasing scientific interest in the use of medicinal plants and plant-derived compounds as complementary or alternative therapeutic strategies. Phytotherapy has been widely employed for centuries in traditional medical systems, such as Ayurveda, where plant-based remedies are used to restore metabolic balance. However, despite their extensive traditional use, only a limited number of medicinal plants have been rigorously validated through experimental and clinical studies [7,8].

To better understand the pathophysiology of diabetes and to evaluate potential therapeutic agents, numerous experimental models have been developed, among which streptozotocin-induced diabetes is one of the most widely used. Streptozotocin selectively targets pancreatic β-cells, leading to insulin deficiency and the establishment of a stable hyperglycemic state, thereby providing a reliable model for investigating both the mechanisms of disease and the efficacy of antidiabetic compounds. The characteristics and severity of the induced diabetic state depend on several factors, including the administered dose, route of administration, and the animal species used [9,10].

A growing body of evidence highlights the role of plant-derived bioactive compounds, particularly phenolic constituents such as flavonoids, anthocyanins, tannins, coumarins, and phenolic acids, in the modulation of glucose homeostasis and oxidative stress. These compounds exhibit potent antioxidant activity, reduce DNA damage, and contribute to the prevention of cardiovascular and neurodegenerative complications associated with diabetes. Moreover, they have been reported to exert multiple pharmacological effects, including antidiabetic, anti-inflammatory, antimicrobial, and photoprotective activities [11,12].

The hypoglycemic action of plant-derived compounds is mediated through several complementary mechanisms. These include the stimulation and regeneration of pancreatic β-cells, leading to enhanced insulin secretion, as well as insulin-mimetic effects through the activation of insulin receptors. In addition, such compounds may reduce intestinal glucose absorption, decrease renal glucose reabsorption, inhibit hepatic gluconeogenesis, and promote peripheral glucose utilization. Improvement of insulin sensitivity and modulation of hepatic glucose metabolism further contribute to their overall antidiabetic effect. Some phytochemicals are also capable of inhibiting insulin-degrading enzymes or reactivating protein-bound insulin, thereby potentiating endogenous insulin activity [13,14,15].

Among medicinal plants with recognized antidiabetic potential, Vaccinium myrtillus has been extensively studied and is widely used as a reference species due to its well-documented hypoglycemic, antioxidant, hypolipidemic, and anti-inflammatory properties. The leaves and fruits of this species contain a complex mixture of bioactive compounds, including flavonoids such as quercetin, anthocyanins (notably delphinidin, cyanidin, petunidin, and malvidin derivatives), tannins, triterpenic acids, and organic acids. These constituents contribute to improved pancreatic vascularization, enhanced mitochondrial function, and modulation of key metabolic pathways. Furthermore, bilberry extracts have been shown to inhibit α-glucosidase activity, thereby reducing postprandial glucose absorption, and to act as agonists of peroxisome proliferator-activated receptor gamma (PPAR-γ), which plays a crucial role in glucose and lipid metabolism [16,17,18,19].

In contrast, *Acanthus balcanicus* remains a relatively underexplored species, with limited available data regarding its phytochemical profile and pharmacological properties. This perennial plant, belonging to the *Acanthaceae* family, is distributed in specific regions of southeastern Europe and has been traditionally associated with various ethnobotanical uses. However, scientific evidence supporting its biological activity, particularly in the context of metabolic disorders, is scarce. This lack of data underscores the importance of investigating its potential therapeutic effects and comparing its activity with that of well-established medicinal plants.

In this context, the present study aimed to provide a preliminary comparative evaluation of *Acanthus balcanicus*, a relatively underexplored species, and *Vaccinium myrtillus*, a better-characterized reference species, in a streptozotocin-induced experimental model of diabetes. While *Vaccinium myrtillus* has been widely investigated for its antioxidant and metabolic effects, limited experimental data are available regarding the phytochemical profile and biological activity of *Acanthus balcanicus* in diabetes-related models. Therefore, this study assessed their phenolic and flavonoid composition, antioxidant activity, metabolic effects, and associated histological changes. The study was not designed to establish a definitive antidiabetic mechanism, but rather to generate preliminary experimental evidence to support future mechanistic and pharmacological investigations. Although *Acanthus balcanicus* was previously investigated by our group in an oral glucose tolerance test performed in mice with normal pancreatic function, that study was limited to the establishment of an effective antihyperglycemic dose. The present study differs by evaluating *Acanthus balcanicus* in a streptozotocin-induced diabetic model and by including metabolic, oxidative stress, and histological endpoints. *Vaccinium myrtillus* was included as a botanical reference comparator because it has been consistently used in our laboratory due to its reproducible hypoglycemic and antioxidant behavior in previous experimental models.

## 2. Results

The quantitative HPLC profile of the selected phenolic compounds identified in the analyzed extracts is presented in Table 1. The results showed that both samples contained detectable amounts of phenolic acids and flavonoid compounds, but with different distribution patterns. In the *Acanthus balcanicus* aqueous extract, the main quantified compounds were chlorogenic acid, quercetin, isoquercitrin, apigenin, apigenin-7-glucoside, and kaempferol. In the *Vaccinium myrtillus* extract, chlorogenic acid, quercetin, and isoquercitrin were present at higher levels compared with ABH, whereas apigenin and kaempferol were more abundant in ABH. Rosmarinic acid was detected in low amounts in both samples) (See Supplementary Materials Figures S1–S5). These data indicate different profiles of selected phenolic markers between the two extracts. However, the relationship between these compositional differences and the in vivo biological effects cannot be directly established from the present data.

For the determination of total phenolic content, the calibration curve obtained using gallic acid was described by the equation y = 0.0023x + 0.0279, with a correlation coefficient of R^2^ = 0.9891, indicating good linearity. Phenolic compounds are known for their antioxidant properties, mainly due to the presence of hydroxyl (-OH) groups, which participate in redox reactions and neutralize reactive oxygen species such as peroxyl, hydroxyl, and alkoxyl radicals. The obtained results demonstrated that both investigated extracts contain appreciable amounts of total phenolic compounds. The aqueous extract of *Acanthus balcanicus* (ABH) showed a slightly higher total phenolic content (0.728 ± 0.067 g GAE/L) compared to *Vaccinium myrtillus* (M-f), which presented a value of 0.682 ± 0.054 g GAE/L. The difference between the two extracts was moderate but not statistically significant (*p* > 0.05), suggesting a comparable phenolic profile. For the determination of total flavonoid content, the calibration curve obtained using quercetin was described by the equation y = 0.0061x + 0.0152, with a correlation coefficient of R^2^ = 0.9988, indicating excellent linearity. Flavonoids are among the most important plant-derived antioxidants, their activity being closely related to their chemical structure and capacity to undergo oxidation reactions. The results showed that *Vaccinium myrtillus* extract exhibited a higher flavonoid content, with a value of 0.765 ± 0.021 g QE/L, compared to *Acanthus balcanicus*, which presented a value of 0.495 ± 0.019 g QE/L, the difference being statistically significant (*p* < 0.01). Regarding pigment composition, both extracts contained measurable amounts of carotenoids and chlorophylls. The *Vaccinium myrtillus* extract exhibited the highest carotenoid content, with a value of 4.62 g/L, while *Acanthus balcanicus* presented a slightly lower value of 4.05 g/L. Chlorophyll a and chlorophyll b were also present in both extracts, with higher concentrations observed in the *Vaccinium myrtillus* extract (chlorophyll a: 0.0271 g/L; chlorophyll b: 0.0689 g/L) compared to *Acanthus balcanicus* (chlorophyll a: 0.0083 g/L; chlorophyll b: 0.0159 g/L). The antioxidant activity of the investigated plant extracts, evaluated by the DPPH radical scavenging assay, revealed significant differences between the tested samples. The aqueous extract of *Acanthus balcanicus* demonstrated a strong free radical scavenging activity, with a mean inhibition percentage of 61.8 ± 3.4%, indicating a high capacity to neutralize DPPH radicals. In contrast, the extract obtained from *Vaccinium myrtillus* exhibited a moderate antioxidant effect, with a mean inhibition value of 52.6 ± 2.8%. Statistical analysis showed that both extracts exhibited significantly higher antioxidant activity compared to the blank (*p* < 0.0001). Furthermore, the difference between the two extracts was also statistically significant (*p* < 0.01), with *Acanthus balcanicus* displaying superior antioxidant potential.

The experimental groups were defined as follows:

Group I, healthy untreated control;
Group II, untreated streptozotocin-induced diabetic control;
Group III, diabetic animals treated with *Acanthus balcanicus* aqueous extract;
Group IV, diabetic animals treated with *Vaccinium myrtillus* aqueous extract.

As shown in Table 2, untreated diabetic animals (Group II) exhibited markedly increased water and food intake compared with the healthy control group (Group I), reflecting the characteristic polydipsia and polyphagia associated with streptozotocin-induced diabetes. Administration of both plant extracts reduced water and food intake in diabetic animals. The effect was more pronounced in Group III, treated with *Acanthus balcanicus*, where water intake decreased to 16.3 ± 3.1 mL/day and food intake to 5.0 ± 0.46 g/day. In Group IV, treated with *Vaccinium myrtillus*, water and food intake were also reduced, reaching 18.4 ± 3.9 mL/day and 5.4 ± 0.81 g/day, respectively. These findings suggest an improvement in diabetes-associated metabolic disturbances following treatment, particularly in the *Acanthus balcanicus*-treated group.

**Figure 1:**
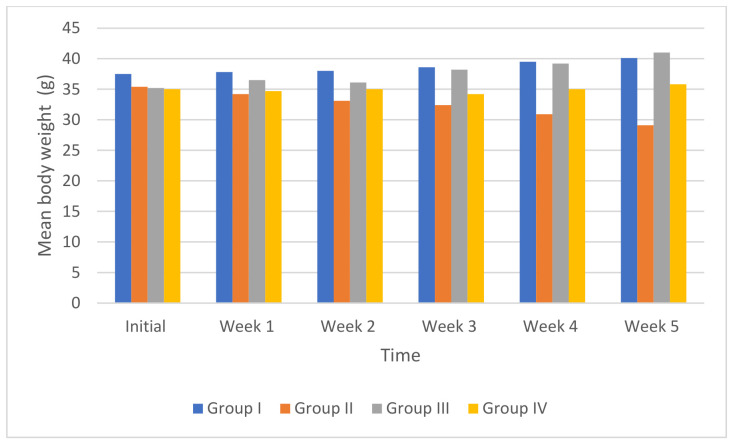
Evolution of mean body weight during the experimental period.

As presented in Figure 1, the healthy control group (Group I) showed a gradual increase in body weight throughout the experimental period, from 37.5 ± 2.1 g initially to 40.1 ± 1.4 g at week 5. In contrast, untreated diabetic animals (Group II) exhibited a progressive decrease in body weight, reaching 29.1 ± 1.5 g at week 5, consistent with the catabolic state associated with streptozotocin-induced diabetes. Treatment with *Acanthus balcanicus* extract (Group III) was associated with a marked improvement in body weight evolution, with values increasing from 35.2 ± 2.0 g initially to 41.0 ± 1.6 g at week 5. The *Vaccinium myrtillus*-treated group (Group IV) also showed stabilization and moderate recovery of body weight, reaching 35.8 ± 1.7 g at week 5. These results suggest that both plant extracts attenuated diabetes-associated weight loss, with a more pronounced effect observed in the *Acanthus balcanicus*-treated group.

**Figure 2:**
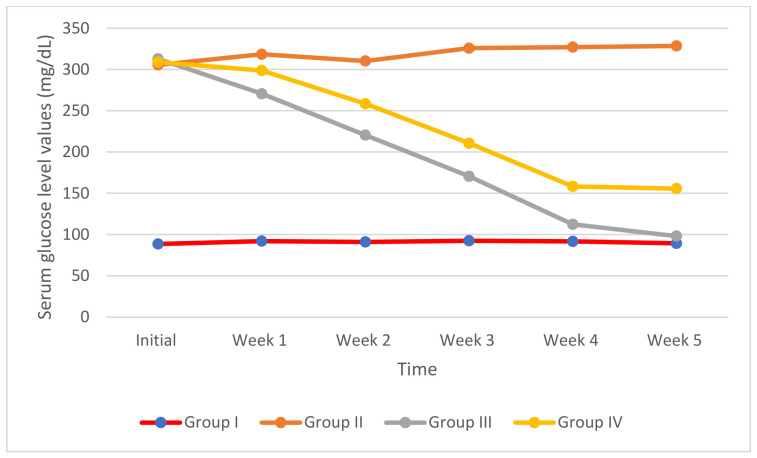
Evolution of serum glucose levels during the experimental period.

The healthy control group (Group I) maintained stable serum glucose levels throughout the experimental period, with values ranging from 88.5 ± 3.0 mg/dL initially to 89.3 ± 2.0 mg/dL at week 5. In contrast, untreated diabetic animals (Group II) showed persistently elevated glucose levels, reaching 328.5 ± 7.2 mg/dL at week 5, confirming the maintenance of streptozotocin-induced hyperglycemia. Treatment with *Acanthus balcanicus* extract (Group III) resulted in a progressive reduction in serum glucose levels, from 312.8 ± 7.5 mg/dL initially to 98.2 ± 7.5 mg/dL at week 5. The *Vaccinium myrtillus*-treated group (Group IV) also showed a gradual decrease in glucose levels, reaching 155.6 ± 6.4 mg/dL at week 5 (Figure 2).

**Figure 3:**
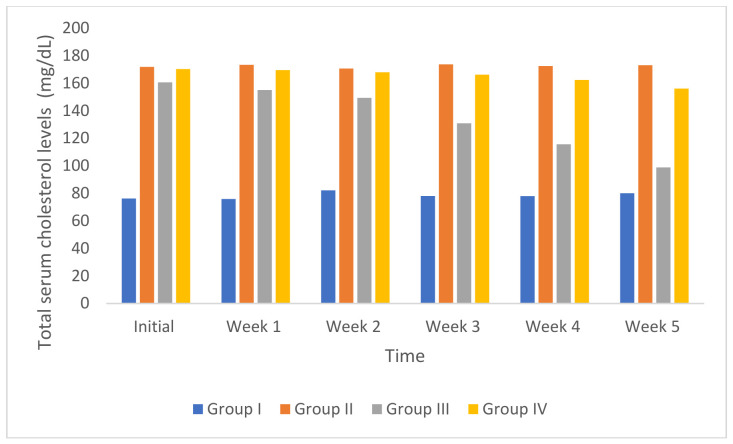
Evolution of total cholesterol levels during the experimental period.

As shown in Figure 3, the healthy control group (Group I) maintained relatively stable total serum cholesterol levels throughout the experiment, with a final value of 80.0 ± 2.6 mg/dL at week 5. In contrast, untreated diabetic animals (Group II) showed persistently elevated cholesterol levels, reaching 173.0 ± 4.2 mg/dL at week 5. Treatment with *Acanthus balcanicus* extract (Group III) resulted in a progressive reduction in total cholesterol, from 160.5 ± 5.2 mg/dL initially to 98.7 ± 5.1 mg/dL at week 5. The *Vaccinium myrtillus*-treated group (Group IV) also showed a decrease in cholesterol levels, although less pronounced, reaching 156.0 ± 4.8 mg/dL at week 5.

Serum triglyceride levels remained stable in the healthy control group (Group I), with values of 109.0 ± 3.1 mg/dL initially and 108.5 ± 2.6 mg/dL at week 5. In contrast, untreated diabetic animals (Group II) showed persistently elevated triglyceride levels, reaching 206.2 ± 5.8 mg/dL at week 5. Treatment with *Acanthus balcanicus* extract (Group III) markedly reduced triglyceride levels from 202.6 ± 5.2 mg/dL initially to 108.9 ± 4.7 mg/dL at week 5, approaching the values observed in the healthy control group. The *Vaccinium myrtillus*-treated group (Group IV) also showed a reduction in triglyceride levels, reaching 150.2 ± 5.4 mg/dL at week 5 (Table 3).

Untreated diabetic animals (Group II) exhibited decreased antioxidant enzyme activities and increased lipid peroxidation compared with the healthy control group (Group I). GR activity decreased from 61.4 ± 2.2 U/L whole blood in Group I to 47.8 ± 2.6 U/L whole blood in Group II, while GPx and SOD activities were also reduced. At the same time, lipid peroxidation increased markedly in Group II, reaching 2.610 ± 0.09 mmol/L, compared with 0.548 ± 0.05 mmol/L in Group I, indicating enhanced oxidative stress under diabetic conditions. Treatment with *Acanthus balcanicus* extract (Group III) improved the antioxidant profile, increasing GR activity to 58.6 ± 1.9 U/L whole blood, GPx activity to 5602.3 ± 210.5 U/L hemolysate, and SOD activity to 197.2 ± 3.1 U/mL hemolysate. Lipid peroxidation was markedly reduced to 0.345 ± 0.04 mmol/L. In the *Vaccinium myrtillus*-treated group (Group IV), GR, GPx, and SOD activities also increased compared with the untreated diabetic group, while lipid peroxidation decreased to 0.612 ± 0.06 mmol/L (Table 4).

One-way ANOVA followed by Tukey post hoc multiple comparison testing was performed for the main metabolic and oxidative stress parameters. Significant overall differences among the four experimental groups were observed for water intake [F(3,36) = 24.16, *p* < 0.0001], food intake [F(3,36) = 39.16, *p* < 0.0001], final body weight [F(3,36) = 122.10, *p* < 0.0001], blood glucose levels at week 5 [F(3,36) = 3221.58, *p* < 0.0001], total cholesterol at week 5 [F(3,36) = 1083.16, *p* < 0.0001], triglycerides at week 5 [F(3,36) = 930.84, *p* < 0.0001], GR activity [F(3,36) = 73.96, *p* < 0.0001], GPx activity [F(3,36) = 392.24, *p* < 0.0001], SOD activity [F(3,36) = 14.80, *p* < 0.0001], and lipid peroxidation [F(3,36) = 2846.13, *p* < 0.0001].

Post hoc analysis showed that, compared with the untreated diabetic group (Group II), the *Acanthus balcanicus*-treated group (Group III) showed significantly lower water intake (*p* < 0.0001), lower food intake (*p* < 0.0001), higher final body weight (*p* < 0.0001), markedly reduced blood glucose levels at week 5 (*p* < 0.0001), reduced total cholesterol (*p* < 0.0001), reduced triglycerides (*p* < 0.0001), increased GR activity (*p* < 0.0001), increased GPx activity (*p* < 0.0001), increased SOD activity (*p* < 0.05), and reduced lipid peroxidation (*p* < 0.0001). These findings indicate a significant improvement in metabolic and oxidative stress parameters in Group III compared with untreated diabetic animals.

The *Vaccinium myrtillus*-treated group (Group IV) also showed significant improvements compared with the untreated diabetic group, including reduced water intake (*p* < 0.001), reduced food intake (*p* < 0.0001), higher final body weight (*p* < 0.0001), reduced blood glucose levels at week 5 (*p* < 0.0001), reduced total cholesterol (*p* < 0.0001), reduced triglycerides (*p* < 0.0001), increased GR activity (*p* < 0.0001), increased GPx activity (*p* < 0.0001), and reduced lipid peroxidation (*p* < 0.0001). The increase in SOD activity in Group IV compared with Group II did not reach statistical significance.

Direct comparison between the two treated groups showed that *Acanthus balcanicus* produced significantly stronger effects than *Vaccinium myrtillus* on final body weight (*p* < 0.0001), blood glucose levels at week 5 (*p* < 0.0001), total cholesterol (*p* < 0.0001), triglycerides (*p* < 0.0001), GR activity (*p* < 0.001), GPx activity (*p* < 0.0001), and lipid peroxidation (*p* < 0.0001). No significant differences between the two treated groups were observed for water intake, food intake, or SOD activity. Overall, the post hoc analysis supports a more pronounced metabolic and antioxidant effect for *Acanthus balcanicus* under the present experimental conditions.

**Figure 4:**
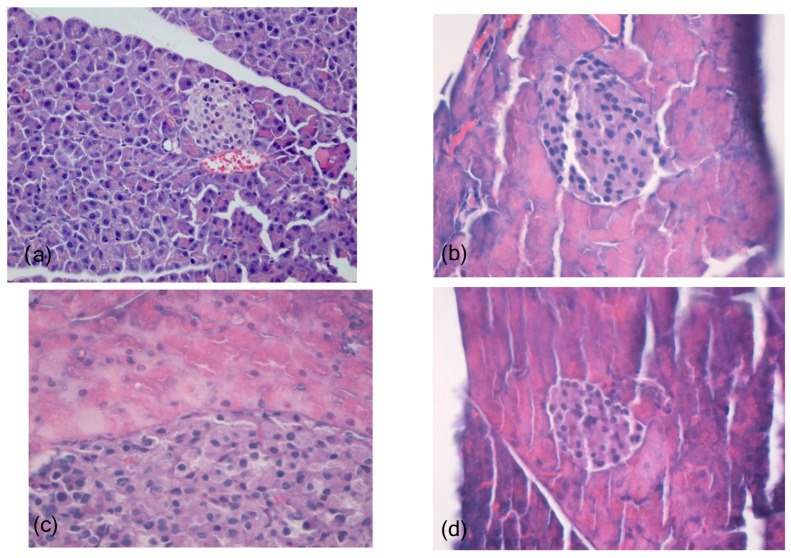
Representative photomicrographs of pancreatic tissue from the experimental groups. (**a**) Representative photomicrograph of the endocrine pancreas from group I, the healthy control group, showing preserved islet architecture and normal pancreatic cytoarchitecture, ×200; (**b**) Group II: islet of Langerhans with necrosis of the adjacent acini, ×400; (**c**) Group III, treated with Acanthus balcanicus herba plant extract: acinar–islet histoarchitecture without structural alterations, ×400. (**d**) Group IV, treated with Vaccinium myrtillus folium plant extract: partially preserved pancreatic architecture due to necrobiotic lesions, with dissociation of acinar areas and insular cords ×400.

Histological examination of biological tissues provided an integrated overview of streptozotocin-induced tissue damage and of the potential protective or reparative effects exerted by the tested plant extracts. In the present study, pancreatic sections from the untreated diabetic group showed marked structural alterations compared with the control group, consisting mainly of necrotic-haemorrhagic lesions, consistent with severe streptozotocin-induced pancreatic injury. In contrast, group III, treated for five weeks with the extract obtained from *Acanthus balcanicus*, showed better preservation of pancreatic cytoarchitecture compared with untreated diabetic animals; however, the descriptive nature of the histological evaluation does not allow definitive conclusions regarding full tissue protection (Figure 4).

**Figure 5:**
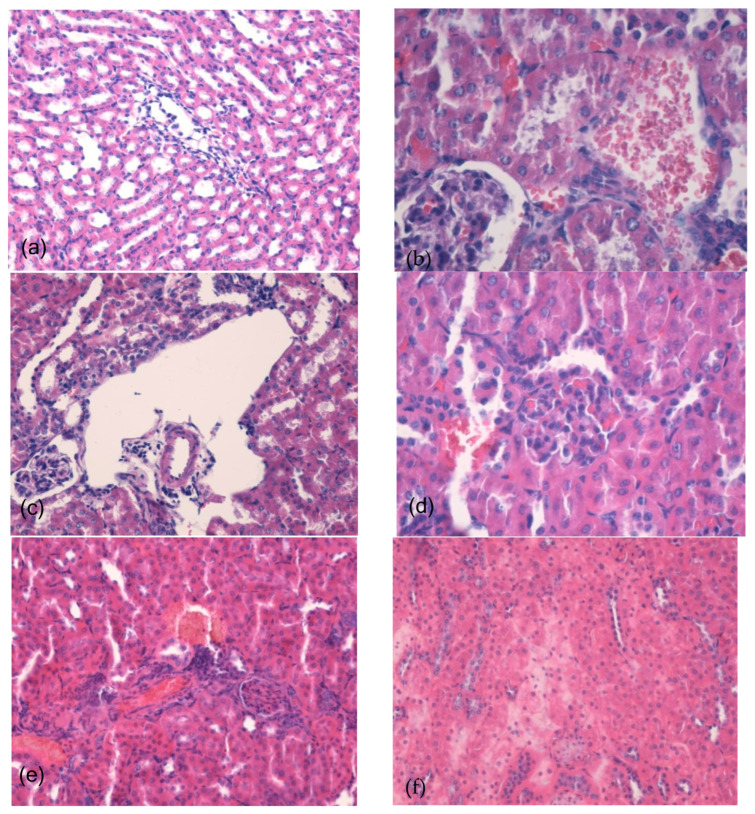

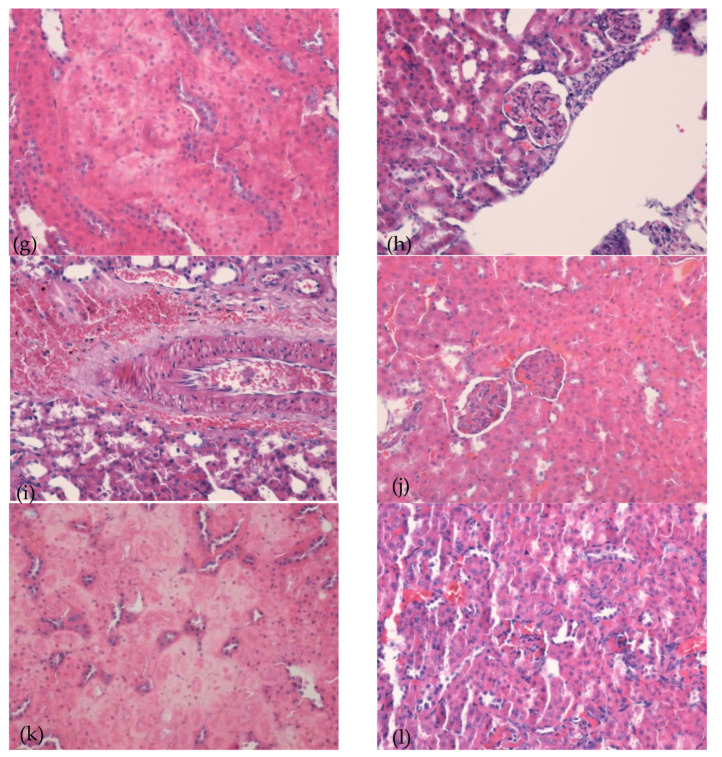
Representative photomicrographs of renal tissue from the experimental groups. (**a**) Group I, healthy untreated control: kidney tissue showing mild diffuse chronic inflammatory infiltrate ×200; (**b**) Group II: renal stasis and haemorrhage associated with degeneration of the tubular epithelium ×400; (**c**) Group II: renal cyst ×200; (**d**) Group II: partially preserved glomerular architecture, obliteration of Bowman’s space, and focal microhaemorrhages ×400; (**e**) Group III, treated with *Acanthus balcanicus herba* plant extract: renal stasis and haemorrhage, ×200. (**f**) Group III, treated with *Acanthus balcanicus herba* plant extract: hyalinization of some renal tubular areas, ×200; (**g**) Group III, treated with *Acanthus balcanicus herba* plant extract: hyalinization of renal tubules, ×200. (**h**) Group III, treated with *Acanthus balcanicus herba* plant extract: hyalinization of the renal tubules, ×400; (**i**) Group IV, treated with *Vaccinium myrtillus folium* plant extract: stasis and haemorrhagic foci in the renal corticomedullary region, ×200. (**j**) Group IV, treated with *Vaccinium myrtillus folium* plant extract: stasis and hyalinization, with renal glomeruli showing markedly dilated Bowman’s space, ×200; (**k**) Group IV, treated with *Vaccinium myrtillus folium* plant extract: vascular stasis and hyalinization, with renal glomeruli showing markedly enlarged Bowman’s space, ×200. (**l**) Group IV, treated with *Vaccinium myrtillus folium* plant extract: microhaemorrhagic foci and isolated thrombosis, with focal dissociative oedema, ×200.

Microscopic evaluation of haematoxylin and eosin-stained renal sections showed that renal parenchymal damage was present in all diabetic groups, with congestion, vascular stasis, and scattered microhaemorrhagic foci observed in both the cortical and medullary regions. Nevertheless, diabetic animals treated with the tested plant extracts—*Acanthus balcanicus* herba and *Vaccinium myrtillus* folium—displayed comparatively better preservation of renal cytoarchitecture. The renal lesions were less extensive and less severe than those observed in untreated diabetic animals, suggesting partial attenuation of renal histological alterations under the present experimental conditions (Figure 5).

**Figure 6:**
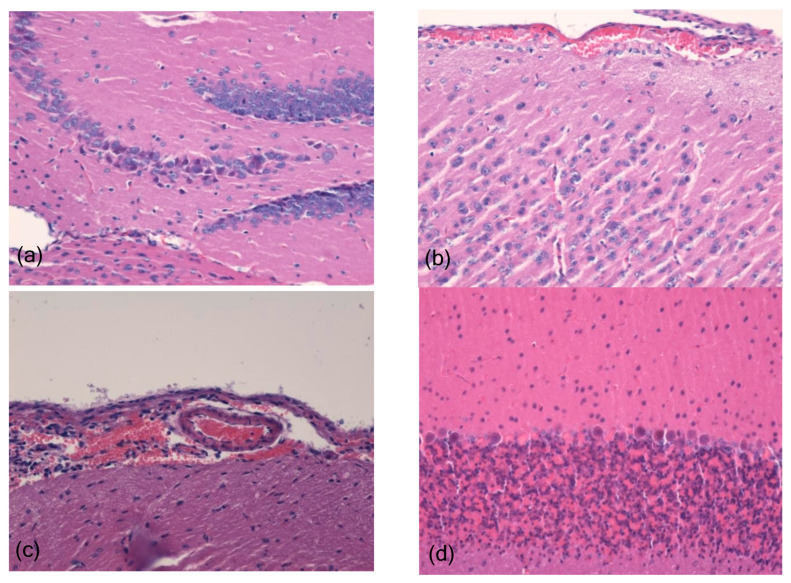

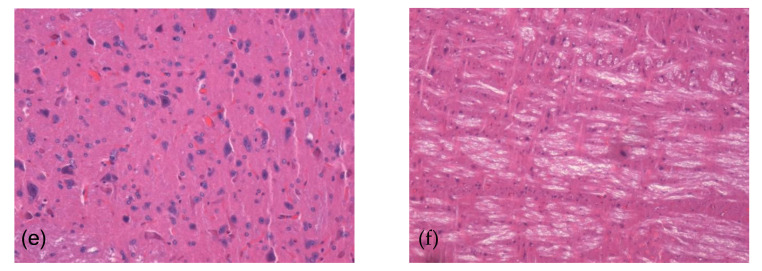
Representative histological aspects of brain tissue in the experimental groups. (**a**) Group I: hippocampus with granular neurons and oedema, ×200. (**b**) Group II: meningeal haemorrhage, ×200; (**c**) Group II: meningeal haemorrhage, ×200. (**d**) Group III, treated with *Acanthus balcanicus herba* plant extract: cerebellar cortex with preserved cytoarchitecture, ×200; (**e**) Group III, treated with *Acanthus balcanicus herba* plant extract: motor nuclei of the brainstem with preserved glioneuronal cytoarchitecture, ×200. (**f**) Group IV, treated with *Vaccinium myrtillus folium* plant extract: rarefaction of the white matter, ×400.

Histological examination of brain tissue was conducted to evaluate the ability of the tested plant extracts to counteract streptozotocin-induced neurohistological damage. In the untreated diabetic group, marked disruption of brain cytoarchitecture was observed, together with meningeal haemorrhage, indicating severe diabetes-associated neurovascular injury. In group IV, corresponding to diabetic animals treated with *Vaccinium myrtillus* folium plant extract, brain sections revealed inflammatory infiltration, vascular stasis, microthrombosis, and white matter rarefaction, suggesting persistent residual neurovascular and parenchymal damage. Among the treated groups, group III, which received *Acanthus balcanicus* extract, displayed preserved brain architecture, comparable to normal histological organization (Figure 6).

In addition to the descriptive histopathological evaluation, a semi-quantitative scoring system was applied to assess the severity of the principal lesions observed in the pancreas, kidney, and brain. This approach provided a standardized comparison of tissue alterations among the experimental groups and facilitated the evaluation of the potential protective effects of the tested plant extracts (Table 5).

## 3. Discussion

Previous investigations performed by our research group on different types of extracts obtained from these plant materials indicated the presence of several classes of bioactive constituents. This previous experience supported the selection of the investigated species and phytochemical markers evaluated in the present study. However, the current analytical assessment was focused on selected phenolic acids and flavonoids quantified by HPLC, together with spectrophotometric evaluation of total phenolic and flavonoid contents. Streptozotocin-induced diabetes mellitus is a well-established experimental model characterized by hyperglycemia, glycosuria, polyphagia, polydipsia, polyuria, marked weight loss, hypoinsulinemia, and hyperlipidemia. Streptozotocin has been widely used in experimental studies due to its selective cytotoxic action on pancreatic β-cells, leading to insulin-dependent diabetes through chemical endocrino-pancreatectomy while preserving exocrine pancreatic function [20,21].

In the present study, untreated diabetic animals (Group II) exhibited a marked increase in water intake, reaching mean values of 31.2 ± 9.4 mL/day, compared to 10.8 ± 3.2 mL/day in the normal control group (Group I), the difference being highly significant (*p* < 0.0001). This reflects the characteristic polydipsia associated with hyperglycemia-induced osmotic diuresis. Treatment with plant extracts significantly reduced water intake, with the most pronounced effect observed in Group III (*Acanthus balcanicus*), where consumption decreased by approximately 48% compared to the diabetic control (*p* < 0.001), indicating a substantial improvement in metabolic regulation.

Similarly, food intake was significantly increased in the diabetic control group (8.7 ± 1.6 g/day) compared to the normal group (4.5 ± 0.52 g/day) (*p* < 0.0001), confirming the presence of polyphagia. Administration of plant extracts resulted in a significant reduction in food intake, particularly in Group III (5.0 ± 0.46 g/day), compared to Lot II (*p* < 0.001), suggesting improved glucose utilization and partial normalization of energy metabolism.

Body weight evolution further supports these findings. Untreated diabetic animals exhibited a progressive and significant decrease in body weight, reaching 29.1 ± 1.5 g at the end of the experimental period, compared to the initial value of 35.4 ± 2.6 g (*p* < 0.0001). In contrast, the normal control group showed a gradual increase in body weight (*p* < 0.05), while the treated diabetic groups exhibited stabilization or moderate weight gain. Notably, animals treated with *Acanthus balcanicus* showed a significant increase in body weight from 35.2 ± 2.0 g to 41.0 ± 1.6 g (*p* < 0.001), suggesting an improvement in metabolic balance under the present experimental conditions. Fasting blood glucose levels clearly demonstrated the diabetogenic effect of streptozotocin. The diabetic control group maintained persistently elevated glycemic values (>320 mg/dL), significantly higher than those of the normal group throughout the experiment (*p* < 0.0001). Treatment with Vaccinium myrtillus resulted in a significant reduction in glycemia from 308.9 ± 6.9 mg/dL to 155.6 ± 6.4 mg/dL (*p* < 0.001 vs. Group II). A more pronounced reduction was observed in Group III, treated with *Acanthus balcanicus*, where glycemia decreased from 312.8 ± 7.5 mg/dL to 98.2 ± 7.5 mg/dL. This decrease indicates a marked improvement in glycemic status compared with untreated diabetic animals; however, the absence of a positive pharmacological control prevents direct comparison with standard antidiabetic therapy.

Lipid profile analysis revealed that diabetic animals exhibited significantly elevated cholesterol levels compared to the normal group (*p* < 0.0001). In the diabetic control group, cholesterol values remained persistently high throughout the experiment. Treatment with Vaccinium myrtillus led to a moderate but significant reduction in cholesterol levels (*p* < 0.01 vs. Group II), while *Acanthus balcanicus* demonstrated a more pronounced hypocholesterolemic effect, reducing cholesterol from 160.5 ± 5.2 mg/dL to 98.7 ± 5.1 mg/dL (*p* < 0.0001 vs. Group II; *p* < 0.01 vs. Group IV). However, cholesterol levels did not fully return to those observed in the normal group (*p* < 0.05).

A similar trend was observed for triglycerides. The diabetic control group maintained elevated triglyceride levels throughout the experiment, significantly higher than the normal group (*p* < 0.0001). Treatment with plant extracts resulted in significant reductions, with Vaccinium myrtillus showing a moderate effect (*p* < 0.01 vs. Group II), while Treatment with *Acanthus balcanicus* was associated with a marked reduction in triglyceride levels, which decreased to 108.9 ± 4.7 mg/dL. These values were significantly lower than those observed in the untreated diabetic group (*p* < 0.0001) and were not statistically different from those recorded in the healthy control group (*p* > 0.05).

Oxidative stress is a key factor in the pathogenesis of diabetes mellitus, resulting from both enzymatic and non-enzymatic mechanisms. Chronic hyperglycemia enhances glucose autooxidation, activates the polyol pathway, and promotes the formation of advanced glycation end-products, all of which contribute to the generation of reactive oxygen species. Additionally, mitochondrial dysfunction further amplifies oxidative stress [22,23].

In this context, antioxidant enzymes such as glutathione reductase (GR), glutathione peroxidase (GPx), and superoxide dismutase (SOD) play a crucial protective role. In the present study, the diabetic control group exhibited significantly reduced GR activity (47.8 ± 2.6 U/L) compared to the normal group (*p* < 0.001). Treatment with Vaccinium myrtillus led to a moderate increase in GR levels (*p* < 0.05 vs. Group II), while *Acanthus balcanicus* produced a highly significant increase (58.6 ± 1.9 U/L; *p* < 0.0001 vs. Group II), approaching normal values.

A similar pattern was observed for GPx and SOD activities. The diabetic control group exhibited significantly reduced enzyme activities (*p* < 0.001 vs. Group I), while treatment with plant extracts significantly improved these parameters. *Acanthus balcanicus* showed the highest GPx activity (5602.3 ± 210.5 U/L), significantly higher than both the diabetic control (*p* < 0.0001) and the Vaccinium myrtillus group (*p* < 0.01). SOD activity was also significantly increased in treated groups compared to the diabetic control (*p* < 0.05–0.01), indicating improved antioxidant defense.

Lipid peroxidation, assessed by malondialdehyde levels, was significantly increased in the diabetic control group (2.610 ± 0.09 mmol/L) compared to the normal group (*p* < 0.0001). Treatment with plant extracts significantly reduced lipid peroxidation, with Vaccinium myrtillus showing a moderate effect (*p* < 0.01 vs. Group II), while *Acanthus balcanicus* demonstrated a highly significant reduction (0.345 ± 0.04 mmol/L; *p* < 0.0001 vs. Group II; *p* < 0.01 vs. Group IV). The biological activity of Vaccinium myrtillus extracts may be associated with the presence of phenolic acids, flavonoids, anthocyanins, and tannins, compounds previously reported to exert antioxidant, insulinomimetic, and antihyperglycemic effects. Similar antioxidant and metabolic protective mechanisms may also contribute to the activity of *Acanthus balcanicus* extracts.

Histological evaluation of the pancreas in laboratory animals with streptozotocin-induced diabetes, particularly mice and rats, commonly reveals a marked reduction in the number and size of the islets of Langerhans, degeneration and morphological alteration of pancreatic β-cells, β-cell aggregation, hydropic degeneration, and necrosis [24]. Experimental studies have reported the regenerative efficacy of plant extracts on pancreatic architecture in animal models of streptozotocin-induced diabetes. Numerous plant-derived products, including extracts obtained from *Callistemon lanceolatus* Merr. (Myrtaceae), *Elephantopus scaber* L. (Asteraceae), *Ephedra distachya* L. (Ephedraceae), *Ficus amplissima* Sm. (Moraceae), *Nymphaea pubescens* Willd. (Nymphaeaceae), and *Phyllanthus niruri* L. (Euphorbiaceae), have been reported to exert regenerative effects on pancreatic β-cells, thereby positively influencing insulin synthesis and release [25].

Okpe Oche and collaborators demonstrated the β-cell reparative potential of a plant extract obtained from Vitex doniana Sweet (Verbenaceae). In untreated diabetic animals, pancreatic tissue exhibited a significant reduction in islet cell size, disruption of pancreatic architecture, mononuclear cellular infiltration, and the presence of sinusoidal spaces. In contrast, histopathological examination of the pancreas from animals treated with Vitex doniana extract revealed restoration of islet size, together with an increase in β-cell density and percentage. Phytochemical analysis of the extract indicated a complex chemical composition, rich in flavonoids, tannins, saponins, anthraquinone derivatives, and resins [26].

Previous histological investigations performed in mice with streptozotocin-induced diabetes have shown that renal tissue develops lesions resembling those observed in human glomerulosclerosis. These alterations include thickening of the glomerular basement membrane, medullary fibrosis, collagen IV deposition, lymphocytic infiltration, arteriolar hyalinization, and widespread tubular necrosis. The progression of glomerulosclerosis, together with impaired renal function, may ultimately lead to renal failure [27,28].

Diabetic nephropathy is associated with structural remodelling of the glomerular extracellular matrix; however, the precise molecular mechanisms responsible for glomerular basement membrane thickening and extracellular matrix accumulation remain incompletely understood. It has been suggested that increased kallikrein and prostaglandin E_2_ production may contribute to renal hyperfiltration and vasodilation, thereby promoting the development and progression of diabetic nephropathy. Diabetes mellitus is also associated with increased cellular production of eicosanoids in renal tissue, including vasodilatory prostaglandins such as prostaglandin E_2_ and prostacyclin, as well as thromboxane A_2_ [29,30].

The high clinical and economic burden associated with periodic dialysis and renal transplantation in diabetic patients with advanced complications has encouraged the search for alternative therapeutic strategies capable of protecting renal tissue and improving renal function. Experimental studies, including investigations performed in 2012 in mouse models of diabetic nephropathy, have reported nephroprotective effects of several plant-derived products from Indian flora. The proposed mechanisms include reduction in renal mucopolysaccharide infiltration, attenuation of glomerular expansion, decreased tubular dilatation, inhibition of lipid peroxidation, and reduction in reactive oxygen species production [30,31].

In this context, the histological assessment of renal tissue in the present study was performed to evaluate whether the tested plant extracts could mitigate diabetes-associated renal injury and support structural preservation or tissue repair. Particular attention was given to glomerular architecture, tubular morphology, vascular congestion, inflammatory infiltration, necrotic lesions, and signs of interstitial fibrosis.

Experimental evidence indicates that streptozotocin-induced diabetes is associated with distinct histopathological alterations in the brain. In the striatum, diabetic animals may exhibit vacuolization within striatal fibre bundles, while ultrastructural analysis has revealed myelin sheath rarefaction, partial demyelination, and axonal degeneration. At the cortical level, reported lesions include rarefaction of the neuropil in the molecular layer, dystrophic pyramidal neurons, and signs of neuronal degeneration. These changes are accompanied ultrastructurally by compromised myelin sheath integrity around axons, loss or fragmentation of dendritic neurofilaments, mitochondrial abnormalities, and aggregation of cellular organelles [32].

These findings support the concept that diabetes-induced hyperglycaemia and oxidative stress may contribute not only to peripheral organ damage but also to central nervous system injury. In this context, plant-derived compounds with antioxidant and anti-inflammatory properties have attracted increasing interest as potential neuroprotective agents. Studies conducted in streptozotocin-induced diabetic mice have shown that *Curcuma longa* L. may attenuate diabetes-associated cognitive impairment and cholinergic autonomic nervous system dysfunction, suggesting a potential protective effect against diabetic neurodegeneration [33].

The histopathological findings provide morphological support for the biochemical improvements observed after treatment with the investigated plant extracts. In the untreated diabetic group, streptozotocin-induced hyperglycaemia and oxidative stress were associated with severe pancreatic, renal, and brain tissue alterations, including necrotic-haemorrhagic pancreatic lesions, renal vascular stasis, tubular epithelial degeneration, glomerular changes, meningeal haemorrhage, and white matter rarefaction. These lesions are consistent with the systemic impact of diabetes-induced oxidative stress and microvascular injury. In contrast, animals treated with *Acanthus balcanicus* showed better preservation of pancreatic acinar–islet architecture, improved renal cytoarchitecture, and preserved glioneuronal organization in the cerebellum and brainstem. These findings are in agreement with the stronger antioxidant and hypoglycaemic effects observed in this group, suggesting that the extract may limit streptozotocin-induced tissue injury through antioxidant, metabolic, and possibly microvascular protective mechanisms. *Vaccinium myrtillus* treatment was also associated with partial tissue preservation, particularly at the pancreatic and renal levels, although residual lesions such as vascular stasis, hyalinization, microhaemorrhagic foci, and white matter rarefaction were still observed. Overall, the histological findings strengthen the biological relevance of the metabolic and oxidative stress results and support the tissue-protective potential of the tested plant extracts, especially *Acanthus balcanicus*.

Several limitations of the present study should be acknowledged. First, the experimental model was based on streptozotocin-induced diabetes, which mainly reflects insulin-deficient pancreatic β-cell injury and does not fully reproduce the complex pathophysiology of type 2 diabetes. Second, no positive pharmacological control was included, limiting the possibility of comparing the efficacy of the extracts with standard antidiabetic drugs. Third, insulin levels, pancreatic functional markers, and tissue-level oxidative stress parameters were not measured. Fourth, although histological evaluation was added, immunohistochemical and morphometric analyses were not performed. Fifth, the study evaluated whole aqueous extracts, and therefore the contribution of individual compounds, including quercetin, kaempferol, chlorogenic acid, and other phenolic constituents, cannot be established. Finally, the short duration of the experiment and the animal-based design limit direct extrapolation to human diabetes management.

## 4. Materials and Methods

### 4.1. Plant Material and Preparation of Aqueous Extract

The plant materials subjected to investigation (herba for *Acanthus balcanicus* and folium for *Vaccinium myrtillus*) were harvested from species cultivated in the Botanical Garden of the University of Craiova during the April–June vegetation period. The plant materials were taxonomically authenticated by Prof. Ing Silvia-Marcela Osiceanu, Department of Pharmacognosy–Phytochemistry–Phytotherapy, “Alexandru Buia” University Botanical Garden. Following collection, the plant material was carefully cleaned and dried under well-ventilated conditions at ambient temperature until a constant weight was achieved, in order to preserve thermolabile phytoconstituents. Subsequently, the dried vegetal products were ground using an electric grinder and passed through a sieve to obtain a coarse powder with a particle size of approximately 1–2 mm. This degree of fineness was selected to ensure homogeneity and adequate solvent penetration during extraction, while maintaining efficient filtration of the aqueous extract. The powdered plant material was mixed with distilled water at a ratio of 1:10 (*w*/*v*) and maintained at room temperature (24 °C) for 36 h, with periodic agitation to facilitate the extraction of water-soluble bioactive compounds. After extraction, the aqueous extracts were first filtered through filter paper to remove plant residues and coarse particulate material. The filtrates were then centrifuged at 3000 rpm for 5 min, and the resulting supernatants were passed through sterile 0.22 µm membrane filters under aseptic conditions. The sterile-filtered extracts were collected in sterile containers, stored at 4 °C, and used shortly after preparation. No long-term storage was performed. The extraction yield was calculated after solvent evaporation and expressed as a percentage (%) relative to the initial dry plant material. The extraction yields were 13.2% for *Acanthus balcanicus* and 15.7% for *Vaccinium myrtillus*, expressed relative to the initial dry plant material. The aqueous extraction approach was selected to obtain polar extracts suitable for oral administration in the experimental model. The present study does not aim to provide a complete phytochemical characterization of all bioactive classes, but focuses on selected phenolic compounds, total phenolic and flavonoid contents, and antioxidant activity assessed by the DPPH assay [34,35]. Reference samples corresponding to the tested plant materials were preserved in the collection of the Pharmacognosy Laboratory, Faculty of Pharmacy, University of Craiova.

### 4.2. HPLC Analysis of Flavonoids and Phenolic Acids

The qualitative and quantitative analysis of flavonoids and phenolic acids present in the plant extracts was performed by high-performance liquid chromatography coupled with UV–diode array detection (HPLC–UV-DAD). The analysis was carried out using a Jasco MD-2015 HPLC system equipped with a dual-pump system, column thermostat, on-line degasser, and UV–DAD detector. Chromatographic separation was performed on a reversed-phase C18 column, 250 × 4.6 mm, 5 µm particle size. The column temperature was maintained at 25 °C.

The mobile phase consisted of acetonitrile as solvent A and 0.1% phosphoric acid in water as solvent B. The flow rate was 1.0 mL/min, and the injection volume was 10 µL. A gradient elution program was applied as follows: initial conditions, 10% A and 90% B; 13.1 min, 22% A and 78% B; 14.1 min, 40% A and 60% B; and 20.1 min, 40% A and 60% B. Detection was performed at 330 nm, a wavelength suitable for the analysis of phenolic acids and flavonoid compounds due to their characteristic UV absorption.

The detected peaks were putatively assigned by comparison of their retention times and UV–DAD spectra with those of authentic reference standards analyzed under identical chromatographic conditions. Because HPLC–UV/DAD does not provide definitive structural elucidation, these assignments should be regarded as tentative and would require confirmation by complementary techniques such as LC–MS/MS or NMR. The following retention times were recorded for the reference compounds: chlorogenic acid, 7.12 min; caffeic acid, 7.96 min; ferulic acid, 13.15 min; rutin, 15.19 min; isoquercitrin, 15.68 min; rosmarinic acid, 17.58 min; apigenin-7-glucoside, 17.65 min; quercetin, 18.71 min; and kaempferol, 20.25 min. Quantification was performed using calibration curves obtained from the corresponding reference standards, and the results were expressed as mg/g dry extract equivalent after normalization to the dry residue content of each extract. The standards were purchased from Sigma-Aldrich/Merck and were of analytical grade, with purity ≥99%, according to the manufacturers’ certificates of analysis. Quantification was performed using calibration curves prepared from the corresponding reference standards. The HPLC procedure was used for targeted quantification of selected phenolic markers and was not intended as a full method-validation study. Therefore, the absence of complete validation parameters, including LOD, LOQ, recovery, and precision for all analytes, is acknowledged as a limitation [36,37].

### 4.3. Spectrophotometric Determination of Total Phenolic, Flavonoid, Carotenoid, and Chlorophyll Contents

The phytochemical composition of the investigated extracts was further assessed by spectrophotometric determination of total phenolic content, total flavonoid content, carotenoids, and chlorophylls. These parameters were considered compositional indicators and were not used as direct measures of antioxidant capacity. Total phenolic content was determined using the Folin–Ciocâlteu method and expressed as gallic acid equivalents, while total flavonoid content was determined by the aluminium chloride colorimetric method and expressed as quercetin equivalents. Carotenoid and chlorophyll contents were determined spectrophotometrically according to the referenced method.

(1) $$\mathrm{Total}\;\mathrm{phenolics}\;(g\;\mathrm{GAE}/L)=\frac{A_{\mathrm{sample}}-b}{a}$$

where $A_{\mathrm{sample}}$ represents the absorbance of the sample, while a and b correspond to the slope and intercept of the calibration curve [38,39].

Total flavonoid content was determined using the aluminum chloride colorimetric method, with quercetin as a standard. Absorbance was measured at 415 nm, and the results were expressed as quercetin equivalents (QE), calculated as:

(2) $$\mathrm{Total}\;\mathrm{flavonoids}\;(\mathrm{mg}\;\mathrm{QE}/L)=\frac{A_{\mathrm{sample}}-b}{a}$$

Carotenoid and chlorophyll contents were determined spectrophotometrically by measuring absorbance at 470, 653, and 666 nm. The concentrations were calculated using the equations proposed by Lichtenthaler and Wellburn:

(3) $$\begin{array}{c} \mathrm{Chlorophyll}\;a\;(\mathrm{mg}/L)=15.65\times A_{666}-7.34\times A_{653}\\ \mathrm{Chlorophyll}\;b\;(\mathrm{mg}/L)=27.05\times A_{653}-11.21\times A_{666}\\ \mathrm{Total}\;\mathrm{carotenoids}\;(\mathrm{mg}/L)=\frac{1000\times A_{470}-2.86\times C_{a}-129.2\times C_{b}}{245} \end{array}$$

where $A_{470}$, $A_{653}$, and $A_{666}$ represent the absorbance values measured at the corresponding wavelengths, while $C_{a}$ and $C_{b}$ correspond to chlorophyll a and chlorophyll b concentrations [40].

All measurements were performed in triplicate, and results were expressed as mean ± standard deviation. These determinations provide essential information regarding the phytochemical composition of the extracts and their antioxidant potential.

### 4.4. In Vitro Antioxidant Activity by DPPH Radical-Scavenging Assay

The in vitro antioxidant activity of the extracts was evaluated using the DPPH radical-scavenging assay, according to the referenced method with minor adaptations. Briefly, the extract solutions were mixed with DPPH solution, incubated in the dark, and the absorbance was measured spectrophotometrically. The radical-scavenging activity was expressed as percentage inhibition of DPPH radicals.

(4) $$\mathrm{DPPH}\;\mathrm{inhibition}\;(\%)=\frac{A_{\mathrm{blank}}-A_{\mathrm{sample}}}{A_{\mathrm{blank}}}\times 100$$

where $A_{\mathrm{blank}}$ represents the absorbance of the control reaction (DPPH solution without extract), and $A_{\mathrm{sample}}$ represents the absorbance in the presence of the plant extract [41,42].

### 4.5. In Vivo Testing

#### 4.5.1. Experimental Animals

Healthy male Swiss albino mice, weighing between 35 and 45 g and aged 6–8 weeks, were obtained from the biobase of the Faculty of Medicine and Pharmacy, Craiova. The animals were acclimatized to laboratory conditions and a standard diet for a period of one week prior to the experimental procedures. The animals were housed in polypropylene cages containing wood shavings as bedding in a well-ventilated room maintained under standard laboratory conditions, with a temperature of 24–28 °C, a relative humidity of 60–70%, and a 12 h light/dark cycle. The animals had free access to a standard laboratory diet containing carbohydrates, proteins, lipids, mineral salts, and vitamins, as well as water ad libitum throughout the experiment. All experimental procedures were conducted in accordance with internationally accepted guidelines for the care and use of laboratory animals, and the study protocol was approved by the Ethics and Scientific Deontology Committee of the Faculty of Medicine and Pharmacy, Craiova.

#### 4.5.2. Protocol for Experimental Induction of Diabetes Mellitus

Prior to the induction of diabetes, the animals were subjected to a fasting period of 12 h, with free access to water to maintain adequate hydration. Fasting was continued for approximately three hours after the administration of streptozotocin. Experimental diabetes mellitus was induced by a single intraperitoneal injection of streptozotocin at a dose of 180 mg/kg body weight, freshly prepared prior to administration. The streptozotocin model used in the present study primarily reflects insulin-deficient experimental diabetes due to pancreatic β-cell injury. Therefore, the findings obtained using this model should not be directly extrapolated to type 2 diabetes or to human diabetes management without further validation. The injected volume did not exceed 1 mL per 100 g body weight per animal. The destruction of pancreatic β-cells induced by streptozotocin is associated with a transient excessive release of insulin, which may result in severe hypoglycemia and potentially fatal outcomes. To prevent this complication, the animals were provided with a 5% glucose solution for 24 h following streptozotocin administration. Body weight and blood glucose levels were monitored before streptozotocin injection, as well as at 72 h and one week post-administration. Blood samples were collected after a fasting period of 12 h for biochemical determinations. Animals that did not reach fasting blood glucose values above 290–310 mg/dL after streptozotocin administration, confirmed by two determinations (at 72 h and one week post-injection), or animals showing severe distress unrelated to the experimental protocol were excluded from the diabetic groups. The final analysis included ten animals per group.

Animals presenting fasting blood glucose levels exceeding 290–310 mg/dL, confirmed by two determinations, were considered diabetic and included in the experimental groups. Blood glucose levels were measured using an eBsensor glucometer (Visgeneer Inc., Hsinchu City, Taiwan), with blood samples collected from the tail vein [43,44].

#### 4.5.3. Experimental Model for Evaluation of Hypoglycemic and Hypolipidemic Effects

The hypoglycemic and hypolipidemic potential of the investigated plant extracts was evaluated using a streptozotocin-induced diabetic mouse model. The study focused on a relatively underexplored species, *Acanthus balcanicus*, while *Vaccinium myrtillus*, a plant species with well-documented hypoglycemic activity, was used as a reference. Four experimental groups were established, each consisting of ten animals, which received daily treatment administered orally by gavage at 9:00 a.m. for a period of five weeks. The group size of ten animals was selected based on previous experimental studies performed by our research group using similar diabetic animal models and plant extracts, and was considered sufficient to detect biologically relevant differences in glycemic and oxidative stress parameters while minimizing animal use in accordance with ethical principles.

Four experimental groups were established, each consisting of ten animals. Group I represented the healthy untreated control group. Group II represented the untreated streptozotocin-induced diabetic control group. Group III consisted of diabetic animals treated with *Acanthus balcanicus* herba aqueous extract. Group IV consisted of diabetic animals treated with *Vaccinium myrtillus* folium aqueous extract. Both extracts were administered orally by gavage once daily for 35 days, at 9:00 a.m., in a final volume of 0.3 mL/animal. The administered dose was 145 mg/kg body weight for both extracts, expressed as dry extract equivalent and calculated according to the dry residue content of each liquid extract. The same dose was used for both extracts to allow comparative evaluation under identical experimental conditions. Dose selection was based on previous dose-finding studies performed by our group using the oral glucose tolerance test in mice with normal pancreatic function, as well as previous tolerability observations in related experimental models. A positive pharmacological control, such as metformin or glibenclamide, was not included in the present preliminary study. This represents an important limitation, as it prevents direct comparison of the plant extracts with established antidiabetic drugs. Future studies will include appropriate positive controls to better define the pharmacological relevance of the observed effects. The administered doses were selected based on previous experimental studies performed by our research group, in which the effective dose range and the acute and subacute toxicity profiles of the investigated extracts were evaluated. Prior to treatment initiation (day 0), all animals were weighed, and fasting blood glucose, total cholesterol, and plasma triglyceride levels were determined. During the experimental period, animals were monitored for body weight, food intake, water consumption, glycemic levels, and lipid profile parameters. At seven-day intervals, measurements were performed at the same time of day, following a 12 h fasting period. Food consumption (g) and water intake (mL) were recorded daily for each group, with intake calculated as the difference between the amount provided and the amount remaining unconsumed. Blood glucose was measured using a glucometer, while plasma cholesterol and triglycerides were determined using an AccuTrend analyzer (Roche Diagnostics GmbH, Mannheim, Germany), with blood collected from the tail vein [45,46]. No significant signs of toxicity, mortality, or behavioral abnormalities were observed during the treatment period.

#### 4.5.4. In Vivo Assessment of Antioxidant Activity

After the five-week treatment period, the animals were sacrificed, and blood samples were collected in heparinized tubes. Plasma was separated by centrifugation, and erythrocytes were subjected to controlled hemolysis for further biochemical analysis. For hemolysis, approximately 0.5 mL of whole blood was centrifuged for 10 min at 3000 rpm, the plasma was removed, and the erythrocytes were washed four times with physiological saline and centrifuged. Hemolysis was induced by adding 2 mL of cold bidistilled water, followed by incubation for 15 min under controlled conditions. The resulting lysate was diluted with 0.01 M phosphate buffer (pH 7) and used for enzymatic determinations. The biochemical investigations included the determination of antioxidant enzyme activities and lipid peroxidation markers. Superoxide dismutase (SOD) activity was measured based on the inhibition rate of the oxidation of a tetrazolium salt (INT) by superoxide radicals generated in the xanthine oxidase reaction system. Glutathione peroxidase (GPx) activity was evaluated by monitoring the change in absorbance at 340 nm associated with NADPH oxidation during the reduction of oxidized glutathione. Glutathione reductase (GR) activity was determined by measuring the decrease in absorbance at 340 nm corresponding to NADPH consumption during the conversion of oxidized glutathione (GSSG) to reduced glutathione (GSH). Lipid peroxidation was assessed by quantifying malondialdehyde, the main product of lipid peroxidation, based on its reaction with 20% thiobarbituric acid, resulting in the formation of a chromogenic complex measurable spectrophotometrically at 532 nm [47,48].

#### 4.5.5. Histopathological Assessment of Biological Tissues from Mice with Experimentally Induced Diabetes Following Five Weeks of Treatment

Histological examination was performed to evaluate the relationship between the hypoglycaemic and antioxidant effects of the plant extracts observed in previous experimental assessments and their potential protective or reparative effects in target organs.

After five weeks of treatment, the mice were deeply anaesthetized by intraperitoneal injection of ketamine/xylazine at doses of 100 mg/kg ketamine and 10 mg/kg xylazine. After confirmation of deep anaesthesia by the absence of pedal withdrawal and corneal reflexes, the animals were euthanized by cervical dislocation, in accordance with the approved institutional animal ethics protocol and current animal welfare recommendations. Death was confirmed by the absence of respiratory movements and heartbeat before tissue collection.

Tissue samples were collected from the brain, kidney, and pancreas and processed for routine histological examination. Briefly, the collected specimens were fixed in 10% neutral-buffered formalin for 24–48 h and subsequently subjected to standard paraffin-embedding procedures. Dehydration was performed by sequential immersion in increasing concentrations of ethyl alcohol. The specimens were immersed twice in 75% ethanol, twice in 90% ethanol, and twice in absolute ethanol, using tightly sealed containers. Tissue clearing was then carried out in xylene to remove residual alcohol and to prepare the specimens for paraffin infiltration. Paraffin impregnation was performed using molten paraffin, which penetrated the tissues and provided a homogeneous consistency suitable for obtaining thin sections. The specimens were subsequently embedded in paraffin blocks using plastic moulds. The paraffin blocks were sectioned using a microtome, and serial sections of approximately 5 μm thickness were obtained. The sections were mounted on clean, degreased glass slides and dried prior to staining. Haematoxylin and eosin staining was performed according to the conventional protocol. The sections were first deparaffinized, rehydrated through graded alcohols, stained with haematoxylin, differentiated in hydrochloric acid, and treated with lithium carbonate solution. Subsequently, the sections were counterstained with eosin, washed in 70% ethanol, dehydrated in absolute ethanol, cleared in xylene, and mounted for microscopic examination. After each step, the slides were briefly rinsed with distilled water. Haematoxylin and eosin staining enabled the identification and evaluation of the main tissue structures based on their differential staining properties. Cell nuclei appeared intensely blue to violet, whereas the cytoplasm exhibited a lighter violet to pink coloration. Collagen fibres were stained pale pink, while elastic and reticulin fibres were not specifically highlighted by this staining method. Histological evaluation of haematoxylin and eosin-stained sections was performed using a ZEISS Axio Imager Z1 light microscope (Carl Zeiss Microscopy GmbH, Jena, Germany) in bright-field mode. The analysis focused on tissue architecture, cellular morphology, vascular changes, inflammatory infiltrates, degenerative lesions, necrotic areas, and possible signs of structural preservation or tissue recovery in the examined organs [48,49]. Histological evaluation was performed by a specialist pathologist blinded to the experimental group allocation. Representative images were selected after examination of multiple fields from each tissue sample.

#### 4.5.6. Statistical Analysis

Data were expressed as mean ± standard deviation. Normality was assessed before statistical testing. For comparisons among multiple groups, one-way ANOVA followed by Tukey’s post hoc test was used. For parameters measured repeatedly over time, repeated-measures ANOVA was applied to evaluate the effects of treatment, time, and their interaction. When assumptions for parametric testing were not met, appropriate non-parametric tests were used. A *p*-value < 0.05 was considered statistically significant. Longitudinal parameters, including body weight and serum glucose levels measured repeatedly over the experimental period, were analyzed using two-way repeated-measures ANOVA, with treatment group and time as factors, followed by appropriate post hoc comparisons. For endpoint parameters measured once at the end of the experiment, one-way ANOVA followed by Tukey’s post hoc test was used. Normality and homogeneity of variance were assessed using the Shapiro–Wilk and Levene tests, respectively. A *p* value < 0.05 was considered statistically significant.

## 5. Conclusions

The present study provides preliminary evidence that administration of aqueous extracts of *Acanthus balcanicus* herba and *Vaccinium myrtillus* folium was associated with improvements in selected metabolic and oxidative stress parameters in streptozotocin-induced diabetic mice. Under the present experimental conditions, *Acanthus balcanicus* showed more favorable changes in some parameters compared with the botanical reference comparator *Vaccinium myrtillus*. However, these findings should be interpreted cautiously because the study did not include a pharmacological positive control, extract-alone groups in healthy animals, multiple doses, insulin or HbA1c measurements, renal and hepatic function markers, or direct molecular validation. Therefore, the results should be considered preliminary and hypothesis-generating rather than definitive evidence of antidiabetic efficacy or mechanism. Further controlled studies are required to confirm these effects and clarify their pharmacological relevance.

## Figures and Tables

**Table 1:** Quantitative profile of selected phenolic compounds in *Acanthus balcanicus* herba and *Vaccinium myrtillus* folium aqueous extracts, expressed as mg/g dry extract equivalent, mean ± SD.

Samples	Chlorogenic Acid [mg/g Dry Extract]	Rosmarinic Acid [mg/g Dry Extract]	Kaempferol [mg/g Dry Extract]	Quercetin [mg/g Dry Extract]	Apigenin [mg/g Dry Extract]	Apigenin-7-Glucoside [mg/g Dry Extract]	Isoquercitrin [mg/g Dry Extract]
ABH	0.0879 ± 0.0114	0.0052 ± 0.0013	0.0364 ± 0.0053	0.0636 ± 0.0076	0.0500 ± 0.0068	0.0417 ± 0.0053	0.0538 ± 0.0068
M-f	0.1605 ± 0.0166	0.0031 ± 0.0009	0.0236 ± 0.0038	0.0898 ± 0.0096	0.0159 ± 0.0032	0.0268 ± 0.0038	0.0790 ± 0.0083

ABH—*Acanthus balcanicus* herba aqueous extract; M-f—*Vaccinium myrtillus* folium aqueous extract. Values were normalized according to the dry residue content of each liquid extract: 13.2% for ABH and 15.7% for M-f.

**Table 2:** Mean water and feed intake for Groups I–IV over the course of the experiment. Data are presented as mean ± SD; *n* = 10 animals/group.

Experimental Group	Water Intake (mL/Day, M ± SD)	Food Intake (g/Day, M ± SD)
Group I	10.8 ± 3.2	4.5 ± 0.52
Group II	31.2 ± 9.4	8.7 ± 1.6
Group III	16.3 ± 3.1	5.0 ± 0.46
Group IV	18.4 ± 3.9	5.4 ± 0.81

**Table 3:** Serum triglyceride levels in the study groups during the experiment, alongside monitoring of water and feed intake (M = mean; SD = standard deviation, *n* = 10 animals/group).

Experimental Group	Initial (mg/dL)	Week 5 (mg/dL)
Group I	109.0 ± 3.1	108.5 ± 2.6
Group II	204.8 ± 6.5	206.2 ± 5.8
Group III	202.6 ± 5.2	108.9 ± 4.7
Group IV	201.4 ± 6.1	150.2 ± 5.4

**Table 4:** Levels of antioxidant enzymes and lipid peroxides in the study groups (M = mean; SD = standard deviation, *n* = 10 animals/group).

Experimental Group	GR (U/L Whole Blood)	GPx (U/L Hemolysate)	SOD (U/mL Hemolysate)	POL (mmol/L)
Group I	61.4 ± 2.2	4320.8 ± 85.6	205.1 ± 2.5	0.548 ± 0.05
Group II	47.8 ± 2.6	3520.4 ± 120.3	188.9 ± 9.8	2.610 ± 0.09
Group III	58.6 ± 1.9	5602.3 ± 210.5	197.2 ± 3.1	0.345 ± 0.04
Group IV	53.8 ± 2.0	4385.6 ± 95.4	193.6 ± 3.8	0.612 ± 0.06

**Table 5:** Semi-quantitative histological assessment of pancreatic, renal, and brain lesions.

Tissue	Parameter	Group I	Group II	Group III	Group IV
Pancreas	Acinar/islet disruption	0	3	1	2
Pancreas	Necrotic/hemorrhagic lesions	0	3	1	2
Kidney	Vascular stasis/hemorrhage	0–1	3	2	2
Kidney	Tubular degeneration/hyalinization	0–1	3	2	2
Brain	Meningeal/neurovascular alterations	0	3	1	2

Scores: 0 = absent; 1 = mild; 2 = moderate; 3 = severe. The assessment was performed descriptively/semi-quantitatively by a histopathologist blinded to treatment allocation.

## Data Availability

To promote transparency and reproducibility, we will provide a detailed data availability statement. The files and data are in the physical and electronic archive of the University of Medicine and Pharmacy Craiova and can be requested from the corresponding author. All data generated or analyzed during this study are included in this article. Further inquiries can be directed to the corresponding author.

## Supplementary Materials

The following supporting information can be downloaded at: https://www.mdpi.com/article/10.3390/ph19071088/s1. Figure S1: HPLC chromatogram for selected flavonoside standards, flavonoid aglycones, and polyphenolcarboxylic acids. Figure S2: HPLC chromatogram for selected flavonoside standards, flavonoid aglycones, and polyphenolcarboxylic acids. Figure S3: HPLC chromatogram for the rosmarinic acid standard. Figure S4: HPLC chromatogram for ABH sample. Figure S5: HPLC chromatogram for VM sample.
