# Supplementary material for: Phytochemical and Preliminary Biological Evaluation of Acanthus balcanicus Aqueous Extract in Streptozotocin-Induced Diabetes
Source: Pharmaceuticals (Basel). 2026 Jul 15;19(7):1088. doi: 10.3390/ph19071088 (PMC13414777; doi:10.3390/ph19071088)
Supplement: Supplementary file 1 [file pharmaceuticals-19-01088-s001.zip › pharmaceuticals-4401086-supplementary/pharmaceuticals-4401086-supplementary.pdf]

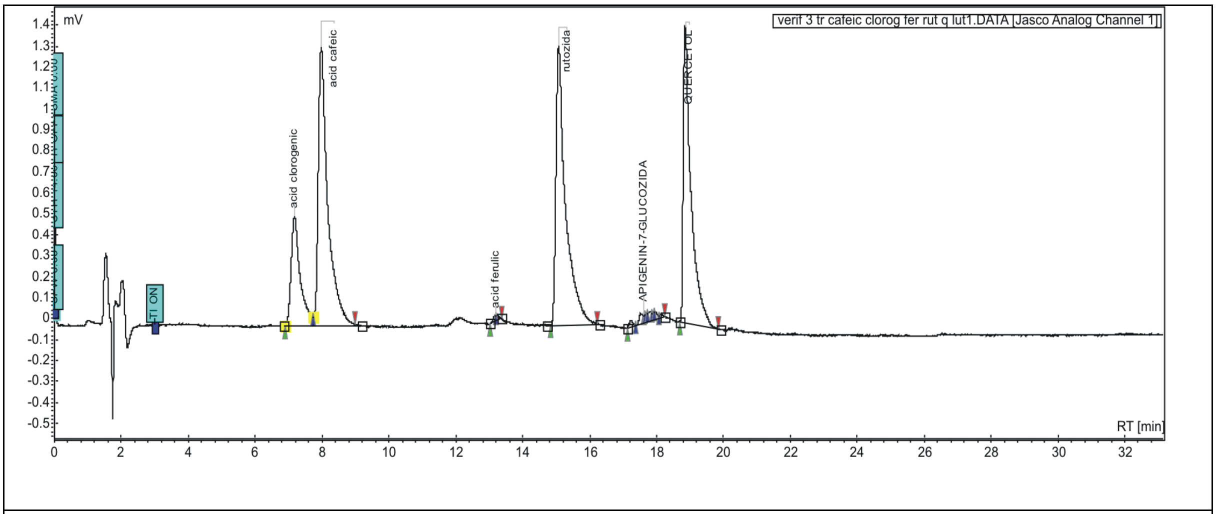

Figure S1. HPLC chromatogram for selected flavonoside standards, flavonoid aglycones, polyphenolcarboxylic acids.

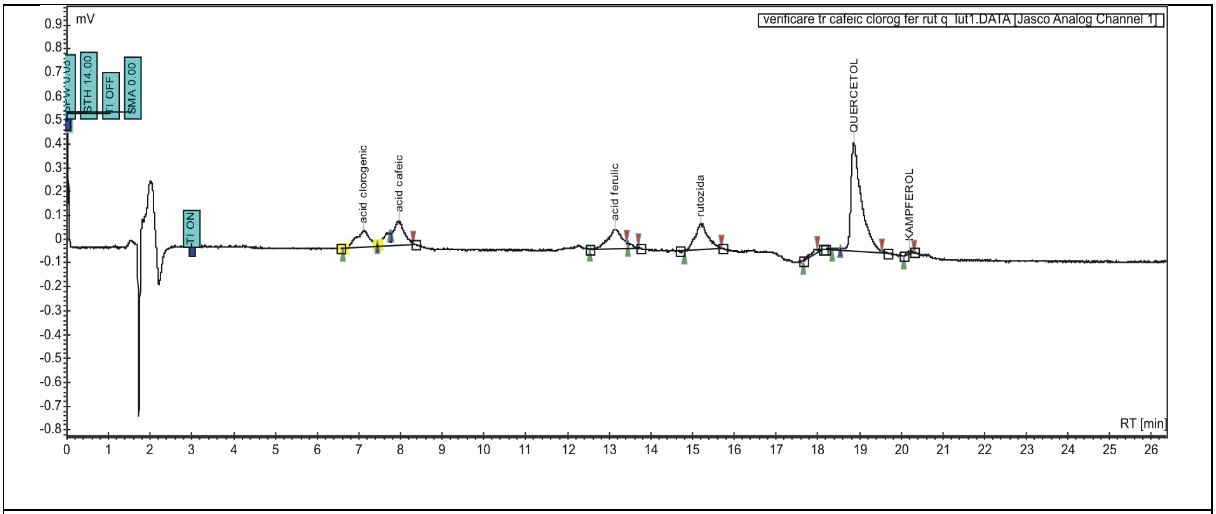

Figure S2. HPLC chromatogram for selected flavonoside standards, flavonoid aglycones, polyphenolcarboxylic acids.

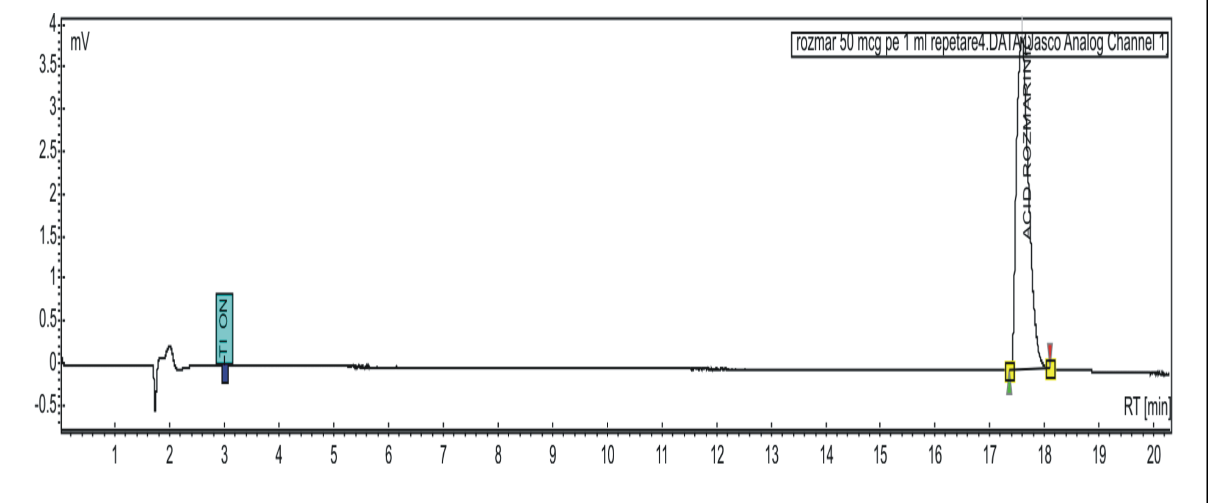

Figure S3. HPLC chromatogram for the rosmarinic acid standard.

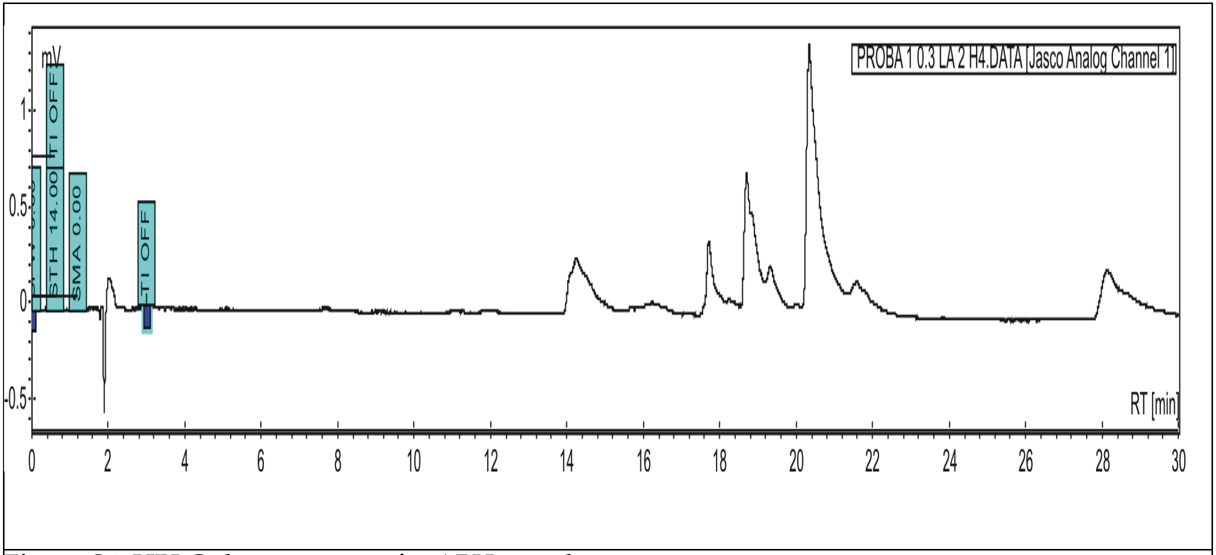

Figure S4. HPLC chromatogram for ABH sample

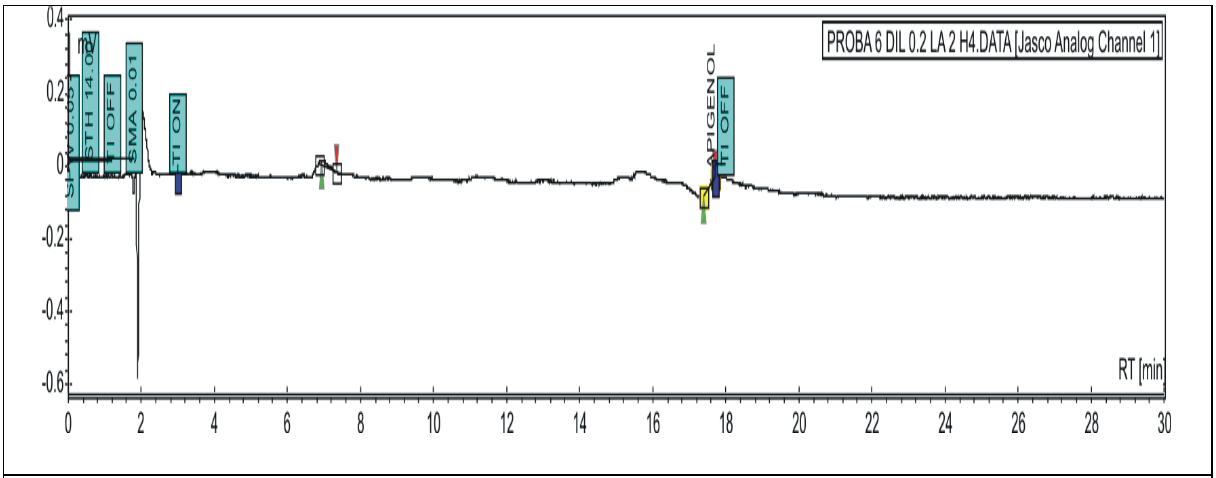

Figure S5. HPLC chromatogram for VM sample
